# Critical Adsorption of Polyelectrolytes onto Patchy Particles with a Low-Dielectric Interior

**DOI:** 10.3390/polym17162205

**Published:** 2025-08-12

**Authors:** Dante A. Anhesini, Daniel L. Z. Caetano, Icaro P. Caruso, Andrey G. Cherstvy, Sidney J. de Carvalho

**Affiliations:** 1 Institute of Biosciences, Humanities and Exact Sciences, São Paulo State University (UNESP), São José do Rio Preto 15054-000, Brazil; dante.anhesini@unesp.br (D.A.A.); daniel.caetano@unesp.br (D.L.Z.C.); icaro.caruso@unesp.br (I.P.C.); 2 Institute for Physics & Astronomy, University of Potsdam, D-14476 Potsdam-Golm, Germany; a.cherstvy@gmail.com

**Keywords:** adsorption of polyelectrolytes, electrostatics, electrostatic potential, low-dielectric interfaces, patchy interfaces, conditions of critical adsorption, polyelectrolyte–protein complexation

## Abstract

A polyelectrolyte (PE) chain in the vicinity of an oppositely charged surface can exhibit a discontinuous transition from the adsorbed to the desorbed state once the electrostatic attractive interactions are not strong enough to overcome the entropic losses caused by the PE-surface adsorption. In the context of PE–protein interactions, the heterogeneity of the charge distribution and the effects of a low dielectric permittivity underneath the surface are crucial. Studies of the combined effects of these two properties are very sparse, especially in the spherical geometry; we thus fill this gap here. We study the adsorption of PE chains onto spherical particles with heterogeneously charged surfaces, with the main focus on the critical-adsorption conditions and the effects of a low-dielectric core. Metropolis Monte Carlo simulations are employed, with the PE exploring the phase-space around the binding particle in the canonical ensemble. Two adsorption–desorption transitions are observed when the particle possesses a net charge of the *same sign* as that of the PE, resulting in *nonmonotonic* behavior of the critical charge density required for the PE–particle electrostatically driven adsorption. An increased affinity between the PEs and low-dielectric particles with variable heterogeneous charge distributions is observed, in contrast to the behavior detected for homogeneous low-dielectric particles. This higher affinity occurs when the Debye screening length in the solution becomes comparable to the dimensions of a patch of the opposite sign to the PE. A number of real-life applications of the considered PE–particle system is presented in the discussion, in particular regarding the properties of the complex formation between various PEs and globular proteins featuring a dipolar-type distribution of electric charges on their surfaces, such as insulin and bovine serum albumin.

## 1. Introduction

### 1.1. Adsorption, Critical Adsorption, and Complex Formation

The adsorption of flexible polyelectrolytes (PEs) [1,2,3,4] onto oppositely charged surfaces has been studied extensively; see the theoretical and computational [5,6,7,8,9,10,11,12,13,14,15,16,17,18,19,20,21,22,23,24,25,26,27,28,29,30,31,32,33,34,35,36,37,38,39,40,41,42,43,44,45,46,47,48,49,50,51,52,53,54,55,56,57,58,59,60,61,62,63,64,65] and experimental [66,67,68,69,70,71,72,73,74,75,76,77,78,79,80,81,82] studies. Often, it exhibits a phase-transition-like behavior when the ionic strength of the solution or the charge density of the surface electric charges reach the values at which the screened electrostatic (ES) attractive interaction energy can no longer overcome the translational and conformational entropic losses caused by the polymer adsorption [41]. In a series of studies over several decades on the PE complexation with various micelles and dendrimers developed and conducted by a group of experts around Dubin [66,67,68,69,70,72,73,74,75,76,77,78,79,80,81], it was shown that the critical conditions for the PE adsorption obey a power law that relates the *critical* (indicated by the subscript “c” hereafter) surface charge density σ and the reciprocal Debye screening length κ, namely [41]

(1) $$\sigma_{c}\left(\kappa \right)∼\kappa^{\alpha },$$

with a geometry-dependent exponent *α*.

Several theoretical approaches have been proposed to evaluate the statistical properties of the adsorbed PE chains and the nature of the PE-surface adsorption–desorption transition [5,6,7,8,9,10,12,14,16,18,24,28,29,36,41,42,43,45,48,53,57,59,62,64,83]. For instance, Cherstvy and Winkler presented in ref. [41] a unified approach for the three basic geometries using the well-known quantum mechanical Wentzel–Kramers–Brillouin (WKB) method [84] to approximately solve the Edwards [85] equation for the PE density profiles and to quantify the PE adsorption onto *homogeneously* charged planar, cylindrical, and spherical interfaces. Note that the properties of the strong PE–surface adsorption—when the PE chain assumes a specific pattern on the oppositely and homogeneously charged surfaces—was also investigated [10,11,23,25,28,36,45]. The results were shown to have a good agreement with the experimental data for the conditions when the surface can be considered to be homogeneously charged. In these three situations, the exponent α assumes the values α={1,2,3} for the spherical, cylindrical, and planar surfaces, respectively. The conditions of the critical adsorption of flexible and worm-like PEs inside the oppositely charged confining cylindrical and spherical cavities were also considered [28,36,42,53,86], with some applications, e.g., to the charge stoichiometry of the nucleic acids and of the capsid proteins for a number of spherical single-stranded DNA and RNA viruses [27,40].

Several computational approaches have been adopted over the last few decades (i) to investigate the effects of important and realistic features not considered in the simplest Gaussian-chain model of PEs, such as a finite length of the PE, a nonuniformity of its charge distribution, a stiffness of the chain, etc., as well as (ii) to verify the description of the PE-surface adsorption provided by the analytically solvable approximate models; see refs. [15,39,46,50,51,52,54,63].

### 1.2. Effects of Charge Heterogeneities and of Dielectric Boundaries

PE adsorption onto *heterogeneously* charged surfaces was also studied (see, i.a., refs. [13,16,17,31,34,35,44,47,56]), and the effects of (i) the charge patterning on the adsorbed PE conformations, (ii) the spatial distribution of monomers near the surface, and (iii) the strength of the adsorption energy onto the critical-adsorption conditions were examined. Particularly interesting are the applications to protein–PE complexes [20,21,26,30,38,49,55,58,79,80], which can occur even between *similarly charged* macromolecules. This phenomenon is often referred to as adsorption on the “wrong side” of the isoelectric point [82,87,88]. Two mechanisms have been proposed to explain such a complexation: (i) the presence of certain protein regions with a charge opposite to that of the PE (so-called “charge patches”), which promote the adsorption for the situations when the local attractive ES interactions [68,89] overcome the repulsive interactions caused by the net protein charge, and (ii) the charge-regulation effects with charge fluctuations of certain titratable groups can lead to the appearance of an induced charge that locally favors the PE–particle adsorption [90,91].

As a consequence of the charge-patch mechanism, some experiments on the interaction between heparin (a polyanion) and insulin (a small globular protein) have showed [76], e.g., a *nonmonotonic* behavior of the critical pH as a function of the ionic strength, in complete distinction to what is observed for a homogeneously charged case. This nonmonotonicity physically occurs because at low salt concentrations—when the Debye length is long compared to the protein dimensions—an increasing ionic strength favors the adsorption of a polyanion due to ES screening of the ES *repulsions* caused by the net charge of the protein. In contrast, when the Debye length is comparable to the protein dimensions, the local *attractive* ES interactions of the PE to the charged patches of the protein become dominant, and the homogeneous-charge behavior is restored [92,93].

To investigate the critical conditions of the PE ES-driven adsorption onto patchy particles more generally, in the past, we have used the Metropolis Monte Carlo method to simulate a PE chain in the presence of a net-neutral dipolar Janus nanosphere [48]. The latter can be seen as a simplified model for a globular protein with two large oppositely charged patches. These simulations [48] have revealed significant differences in the conditions of critical adsorption, as compared to those of the PEs onto a homogeneously charged sphere [41]. Generally, the critical-adsorption studies between charged protein-like patchy particles and PE chains are rather scarce; we try to fill this gap in the current study.

For the protein–PE interactions, the macromolecular dielectric permittivity becomes important due to a low polarizability of the protein interior, in comparison to reorientations of the water molecules in an aqueous environment [94]. Some studies have examined the PE adsorption in the *presence* of low-dielectric substrates [37,43,60,61], a relevant scenario not only for the PE–protein complexation, but also for the PE deposition onto materials such as mica and silica [71]. In the presence of dielectric boundaries, the PE adsorption onto an uncharged planar [61] and spherical surface [37] or onto surfaces with homogeneous charge distributions [22,43] was considered. In this last scenario, the image-charge repulsion [95] causes an *exclusion* of PEs from the vicinity of homogeneously charged surfaces, as predicted from the solution of the Edwards equation in the so-called Debye-Hückel approximation [96] of the linear ES [43,97] and by the Monte Carlo simulations in the absence of added salt [22].

The effects of both a variable surface-charge distribution and a different dielectric-permittivity value was recently studied by Wang and collaborators, who adopted a planar surface with a discrete-charge distribution [60]. However, the adsorption–desorption critical conditions were not explicitly addressed in ref. [60]. Here, the dielectric discontinuities were also shown to *enhance* the PE adsorption onto surfaces with discrete—rather than uniform—charge distributions [60]. Despite of these efforts, systematic studies addressing the combined and synergistic effects of heterogeneous/arbitrary charge distributions and of variable low-dielectric constant in the interior of spherical particles on the properties of PE adsorption in general and—most importantly from the perspective of critical phenomena—onto the PE-surface adsorbed–desorbed transition are rare in the literature.

Therefore, to obtain a more complete picture of PE adsorption onto protein-like patchy spherical particles, it is important to consider the effects of variable and jump-wise changing dielectric permittivity on a macromolecular scale. The main goal of the current work is, therefore, to generalize the critical conditions for the PE adsorption onto spherical particles by considering (as a concrete example) a heterogeneous charge distribution formed by two oppositely charged symmetric regions/caps with an arbitrary charge density and variable dimensions.

For the current study, the problem is formulated such that a spherical particle with an *arbitrary* surface-charge distribution and with a *low-dielectric* core is considered as an attractive nucleus for adsorption of a PE chain, the critical-adsorption conditions of which need to be examined. To the best of our knowledge, this critical-adsorption problem has never been posed and solved previously in full, and the solution to it regarding the most important critical-adsorption demarcation curve as a function of the added simple salt is not known. This is the main novelty aspect of the current study.

Prior to performing the computer simulations, some nontrivial analytical calculations of the ES potential for an *arbitrary* distribution of the surface charges and in the presence of an *arbitrary* dielectric constant inside the core of a spherical particle need first to be performed. Despite certain similarities with the previous work of the authors on PE-Janus-particle adsorption [48], these cap-type charge distributions are chosen here for illustrative purposes and also as a point of reference (to be able to quantify the differences in the obtained results). Therefore, the novelty lies in the *simultaneous* consideration of the combined effects of an arbitrary charge distribution, a low dielectric constant inside the particle, and the spherical geometry of the adsorbing particle on the statistical properties of the adsorbed PE chain in general and on the location of the adsorption–desorption transition for a realistic choice of the model parameters (relevant to some real-world-conditions) in particular. The latter are, e.g., the critical conditions of adsorption of PEs onto multiple kinds of PE-binding proteins; see, e.g., Ref. [76].

### 1.3. Plan of This Paper

**Figure 1:**
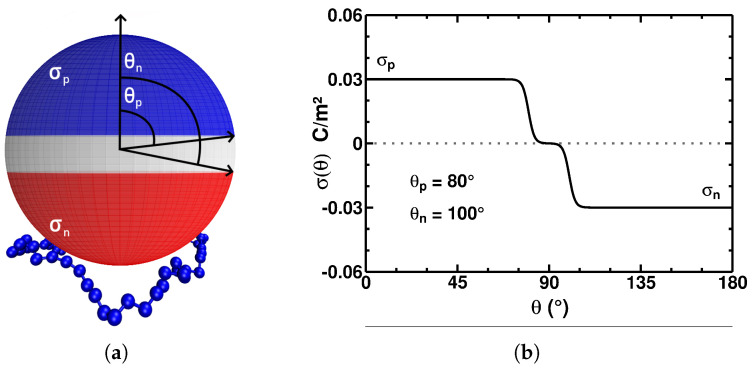
(**a**) Illustrative representation of a spherical particle, with the blue, red, and white colors indicating the positive, negative, and neutral regions, respectively. The positively charged PE chain is illustrated as being adsorbed on the negatively charged cap of this Janus-type [48] particle. (**b**) A plot of Equation (2) representing the surface charge density of the particle with the following model parameters: the angles are $\theta_{p}=80^{\circ }$ and $\theta_{n}=100^{\circ }$, and the surface charge densities are $\sigma_{p}=\sigma_{n}=0.03$ C/m^2^.

In the current study, we consider the properties of critical PE adsorption onto a surface of a low-dielectric spherical particle/protein with a *potentially arbitrary* distribution of electric charges on its surface. As the initial approximation for a patchy distribution of charges, we employ the model of a spherical particle with dielectric discontinuity on the surface and with two oppositely charged hemispheres, which might be separated by a neutral region (a modified Janus-type [48] particle; see Figure 1). Namely, we divide the sphere into three regions with the (correspondingly) positive, zero, and negative charge densities, controlling the size and charge density of these patchy regions in simulations. Similar to our previous studies [48,50], we employ extensive Monte Carlo computer simulations to uncover the critical-adsorption conditions of a PE as functions of (i) the charge density of the patches, (ii) the size of the patches, and (iii) the salinity of the surrounding solution. A comparison of these new findings with the critical-adsorption conditions of a single PE onto a uniformly charged sphere [41] with no dielectric jump is presented too.

This paper is organized as follows. In Section 2, we outline in detail the physical model used in the simulations and discuss the approximations employed, we present the main equations of the model, and we outline the calculations for the distribution of the ES potential. In Section 3, we present the results of the simulations and some underlying data trends of the statistical analysis. Finally, we conclude in Section 4 by providing an overview of possible experimental data where the current results are relevant and by mentioning possible future developments of the proposed model.

## 2. Model and Simulations

### 2.1. Main Equations for the ES Potential

To study the PE adsorption onto a spherical patchy particle, we adopt a coarse-grained model based on a continuum description of the solvent, the particle interior, and on the Debye–Hückel approximation to describe the ES interactions at a given ionic strength [48]. We consider a spherical simulation box containing a free PE and a patchy particle of a fixed radius a=70 Å permanently located at its center. The simulation box is large enough not to affect the polymer conformations and the adsorption properties to be extracted from the simulation data.

We adapt this model for a particle with the charge density depending on the polar coordinate 0≤θ≤π as follows:

(2) $$\sigma \left(\theta \right)=\frac{\sigma_{p}}{2}[1-\tanh(n(\theta -\theta_{p}))]-\frac{\sigma_{n}}{2}[1+\tanh(n(\theta -\theta_{n}))].$$

The surface of the particle for the angles (i) $0\leq \theta \leq \theta_{p}$ has the charge density $\sigma_{p}$, (ii) $\theta_{n}\leq \theta \leq \pi$ has the charge density $-\sigma_{n}$, and (iii) $\theta_{p}\leq \theta \leq \theta_{n}$ has a neutral charge density; see Figure 1. Here, the indices “*p*” and “*n*” in $\sigma_{p,n}$ (which are both positive based on the construction of Equation (2)) correspond to the properties of the positive and negative spherical caps, respectively. The special case of $\theta_{p}=\theta_{n}=\pi /2$ produces the so-called “Janus particle”; we studied the conditions of the critical PE adsorption onto such net-neutral particles without a dielectric-constant jump previously in ref. [48].

The *dimensionless* ES potential Ψ is defined via the standard ES potential ϕ as

(3) $$\Psi =e_{0}\phi /\left(k_{B}T\right).$$

Here, $e_{0}$ is the elementary electric charge and $k_{B}T$ is the thermal energy. The potential $\Psi_{\sigma }$ of a patchy particle is given via an infinite series in terms of the modified spherical Bessel functions of the second kind $k_{l}\left(x\right)$ and of the Legendre polynomials $P_{l}\left(\cos\theta \right)$ [98]; see Appendix A for the details (and also ref. [28] for similar expansions for other heterogeneous patterns of charges on a sphere). Below, we consider the first eleven terms of this series (sufficient for all situations and conditions considered below), i.e.,

(4) $$\Psi_{\sigma }\left(r,\theta \right)=\frac{e_{0}}{2\epsilon_{0}k_{B}T}\sum_{l=0}^{10}\alpha_{l}k_{l}\left(\kappa r\right)P_{l}\left(\cos\theta \right),$$

where $\epsilon_{0}$ is the dielectric permittivity of the vacuum, and the coefficients $\alpha_{l}$ are expressed as follows:

(5) $$\alpha_{l}=\frac{(2l+1)a}{\epsilon_{\mathrm{part}}lk_{l}\left(\kappa a\right)-\epsilon_{\mathrm{water}}\kappa ak_{l}^{\prime }\left(\kappa a\right)}\int_{0}^{\pi }\sigma \left(\theta \right)P_{l}\left(\cos\theta \right)\sin\theta d\theta .$$

Here, $\epsilon_{\mathrm{part}}$ and $\epsilon_{\mathrm{water}}$ are the values of the dielectric constant of the particle interior and of the electrolyte solvent, respectively; $k_{l}^{\prime }\left(x\right)=dk_{l}\left(x\right)/dx$ denotes the standard derivative. The indices “part” and “water” are related to the interior of the particle and to the aqueous solution, respectively. We also use the standard notations for the inverse Debye screening length,

(6) $$\kappa =\sqrt{8\pi l_{B}n_{0}},$$

and for the Bjerrum length in the electrolyte solution,

(7) $$l_{B}=e_{0}^{2}/\left(4\pi \epsilon_{0}\epsilon_{\mathrm{water}}k_{B}T\right).$$

We thus consider an aqueous solution containing the monovalent anions and cations with the number density $n_{0}$. For simplicity, below, we neglect the dependence of $\epsilon_{\mathrm{water}}$ on $n_{0}$; typically, $\epsilon_{\mathrm{water}}\left(n_{0}\right)$ is a decreasing function of $n_{0}$ because of a progressively disrupted ability of interconnecting hydrogen bonds to be created in salty water at high concentrations of the added salt [99,100,101].

The model parameter n=20 specifies the “abruptness” of the charge-density variation in the patch boundary (see Figure 1b); its value was chosen so that the integral of Equation (5) does not measurably change as *n* increases. Note that smooth variations in the charge-density profiles eliminate some problems possibly occurring for the jumps in the charge-density profiles when computing the coefficients in the corresponding eingenfunction expansions.

The PE chain is modeled as a set of N=50 spherical beads of radius b=2 Å (the PE length is fixed hereafter), each with a monovalent positive charge. The beads are linearly connected by the harmonic potential $u_{r}\left(r\right)=k_{r}{\left(r-r_{0}\right)}^{2}$, where the equilibrium distance is set to $r_{0}=7$ Å and the spring constant is $k_{r}=0.5$ N/m (see also our previous simulations-based studies [48,50,57]). The ES interaction energy between the *i*th and *j*th beads on the PE separated by a distance $r_{ij}>4$ Å is given by a screened Coulomb or Yukawa-type [102] potential (computed along the polymer chain):

(8) $$u_{\mathrm{PE}\text{-}\mathrm{PE}}\left(r_{ij}\right)=\frac{l_{B}}{r_{ij}}\exp\left(-\kappa r_{ij}\right).$$

This summation of the Debye–Hückel intercharge potentials thus properly accounts for the ES persistence length of the PE chain in this approximation of exponentially screened ES interactions [25,103]. This level of polymer coarse-graining is rather standard in the PE–surface adsorption studies; for a PE in front of a oppositely uniformly charged surface, it does produce the correct scaling relation for $\sigma_{c}\left(\kappa a\right)$ at κa≫1 [48,57,63]. We thus expect this polymer model to also work well here. In the following, we thus employ the theory of a continuum linear-PB ES and of a continuum dielectric medium. Note also that the performance of the linear- versus nonlinear-PB equations regarding the calculation of the ES potential and the subsequent effects of the potential differences on the location of the adsorption–desorption curve were carefully investigated by the authors previously [50].

Although the Debye–Hückel approximation enables us to calculate the above-mentioned ES potentials analytically, this benefit comes at a certain “cost”. It is well known that this approach is only appropriate at low ES potentials (with a magnitude considerably smaller than about 25 mV). This is valid close to the critical-adsorption conditions, where the adsorbed and desorbed states coexist. For higher magnitudes of the ES potentials, the Debye–Hückel approximation leads to *stronger* adsorption compared to the adsorption based on the accurate ES potential obtained, e.g., via the approximate solutions of the nonlinear PB equation [50]. Furthermore, the nonlinear dependence of the surface charge density on the ES potential at the surface leads to a higher limiting value of κ, beyond which the adsorption is not observed at all [50].

Beyond the mean-field approaches, the incorporation of explicit ions into the simulations would make it possible to evaluate the entropic contributions related, i.a., to the release of condensed/bound counterions [49], which are not being considered here. The effects of ion–ion correlations beyond the mean field are known to be significant, especially for multivalent ions and highly charged surfaces [104,105,106,107,108].

The ES interaction energy between the *k*th bead of the PE at position $\vec{r_{k}}=\left(r_{k},\theta_{k}\right)$ and the patchy particle is given as follows [109] (expressed in units of $k_{B}T$):

(9) $$u_{\mathrm{PE}\text{-}\mathrm{part}}\left(r_{k},\theta_{k}\right)=\Psi_{\sigma }\left(r_{k},\theta_{k}\right)+\frac{1}{2}\Psi_{k,\mathrm{polar}}\left(r_{k},\theta_{k}\right)+\sum_{\begin{array}{c} i=1,i\neq k \end{array}}^{N}\Psi_{i,\mathrm{polar}}\left(r_{k},\theta_{k}\right).$$

Here, the first term describes the direct ES interactions via the potential (4), the second term refers to the polarization charges generated by the *k*th bead close to the dielectric interface, and the third term refers to the polarization charges generated by the presence of all others beads of the PE. The potential $\Psi_{j,\mathrm{polar}}\left(r,\theta \right)$ of the polarization charges (image charges, reaction field) caused by the presence of the *j*th monomer located at position $\vec{r_{j}}=\left(r_{j},\theta_{j}\right)$ is derived in Appendix B and is given as follows:

(10) $$\Psi_{j,\mathrm{polar}}\left(r,\theta \right)=\sum_{l=0}^{10}\frac{e_{0}^{2}\kappa }{4\pi \epsilon_{0}\epsilon_{\mathrm{water}}k_{B}T}\beta_{l}\left(r_{j}\right)k_{l}\left(\kappa r\right)P_{l}\left(\cos\theta_{rj}\right),$$

where the coefficients $\beta_{l}$ are given in terms of the modified spherical Bessel functions of the first kind $i_{l}\left(x\right)$ as follows:

(11) $$\beta_{l}\left(r_{j}\right)=\left[\frac{\epsilon_{\mathrm{water}}\kappa ai_{l}^{\prime }\left(\kappa a\right)-\epsilon_{\mathrm{part}}li_{l}\left(\kappa a\right)}{\epsilon_{\mathrm{part}}lk_{l}\left(\kappa a\right)-\epsilon_{\mathrm{water}}\kappa ak_{l}^{\prime }\left(\kappa a\right)}\right]\left(2l+1\right)k_{l}\left(\kappa r_{j}\right).$$

The ES-potential calculations above are developed to provide a general description applicable to other setups, including others definitions of σ(θ) based on a specific system. In all the results presented here, we assume $\theta_{p}=\theta_{n}$, meaning that no neutral region between the positive and negative patches exists. This is the first approximation to generalize the PE adsorption onto the Janus particles, studied previously [48]. The current model enables us to describe the situations ranging from a homogeneously charged surface to a spherical interface via controlling the geometry of the oppositely charged regions by varying only a few model parameters. This allows us also to evaluate the critical-adsorption conditions for the situation when the particle has a net charge similar to that of the adsorbing PE chain, when the PE is locally attracted to the particle via attractive ES contributions.

### 2.2. Details of Computer Simulations

The computer simulations are performed using the Metropolis Monte Carlo method to sample the phase space, generating a myriad of different configurations of the PE chain through all allowed monomer movements. Specifically, the pivot and crankshaft movements (see ref. [110]), along with translational and rotational motions of the entire chain, as well as arbitrary monomer displacements, are employed in this sampling process.

The simulations consist of two steps. First, various configurations of the polymer are generated to bring the system to a state of equilibrium. The system is considered to be in equilibrium when the total energy fluctuates around its mean value; this state is achieved after ∼$10^{6}$ moves in the simulations. In the second step, a large number of configurations are generated to estimate all relevant statistical averages. To ensure the independence between consecutive configurations, they are separated by $10^{3}$ moves in the simulations. All simulations are performed at a room temperature of T=298.15 K and ∼$10^{7}$ PE configurations were used to estimate the statistical averages.

In the course of the equilibration process, the computer simulations begin with a PE chain in a random configuration located at a random position inside the simulation box. The amplitude of the movements is large to ensure no prolonged localization of the polymer in one of possible metastable states. After the equilibration is complete, the simulations are performed using the pivot and crankshaft movements, and monomer displacements in order to sample all available configurations of the PE in space. The amplitude of these movements is defined by an acceptance of the randomly generated polymer configurations of about 50%. These implemented movements are broadly recognized in the simulation community as well-suited to guarantee a good sampling of the available polymer configurations. Along with these movements, the translational motion of the entire PE chain with a high amplitude is used to promote the “take off” the PE from possible metastable states or to promote a transition between different states of this kind (in particular, between the adsorbed and desorbed states).

As mentioned after Equation (8), the ES contribution to the PE’s length of persistence $l_{p}=l_{p,\mathrm{ES}}+l_{p,\mathrm{non}\text{-}\mathrm{ES}}$ is automatically accounted for in our current simulations. The non-ES contribution to the PE persistence is, however, neglected, as in a number of our previous studies on the critical PE-surface adsorption, both simulations- and theory-based. For the current setup, such a supposition is quite realistic: the PE chains involved in the formation of polymer–proteins complexes are usually very soft [76], with $l_{p}∼10\ldots 40$ Å. If necessary—e.g., for some particular PEs and specific adsorption conditions—the effects of the “structural” mechanical non-ES persistence can be easily incorporated into our future specialized studies of the critical adsorption of flexible-to-semiflexible PE chains.

The statistical deviations from the mean values of the binding energy $\langle E_{B}\rangle$ and of the radius of gyration $\sqrt{\langle R_{g}^{2}\rangle }$ were calculated by dividing the full set of 10^7^ simulated polymer configurations into 10 sections, each with 10^6^ configurations. The mean binding energies are then calculated for all these sections separately and then used to compute the standard deviation $\sqrt{\langle E_{B}^{2}\rangle -{\langle E_{B}\rangle }^{2}}$. For most of the results presented below, these deviations result in the error bars around $\langle E_{B}\rangle$, which are comparable to or smaller than the symbol size used (also for uncertainties of determination of $\sqrt{\langle R_{g}^{2}\rangle }$; thus not shown).

To calculate the values of $\sigma_{n,c}$, we carry out computer simulations for a number of systematically separated values of the charge density $\sigma_{n}$ in a range that ensures that the fraction of polymer configurations in the adsorbed state varies between 0 and 1. We then fit the dataset of the fraction of PE configurations in the adsorbed state versus σ, denoted as $f_{\mathrm{ads}}\left(\sigma \right)$, with a sigmoidal function $f_{\mathrm{ads}}\left(\sigma \right)=\frac{1}{1+e^{\alpha (\sigma -\sigma_{1/2})}}$ with the fit parameters α and $\sigma_{1/2}$. The parameter $\sigma_{1/2}$ then corresponds to the critical values $\sigma_{n,c}$ presented in the plots below for each set of the model parameters. The same procedure is adopted for calculating the values of $\kappa_{c}$. For all critical properties calculated in this study, the correlation coefficient assumes the values ≳0.9.

## 3. Main Results

### 3.1. PE–Particle Binding Energy

Firstly, we analyze the influence of the charge distribution on a particle on the properties of the PE adsorption *without* considering a dielectric interface (no jump of the dielectric constant). We examine the mean binding energy

(12) $$\langle E_{B}\rangle =\langle \sum_{k=1}^{N}u_{\mathrm{PE}\text{-}\mathrm{part}}\left(r_{k},\theta_{k}\right)\rangle$$

as a function of the salt concentration to understand how the range of ES interactions determines the results of the competition between the net- and local-charge density of our Janus-type particle.

**Figure 2:**
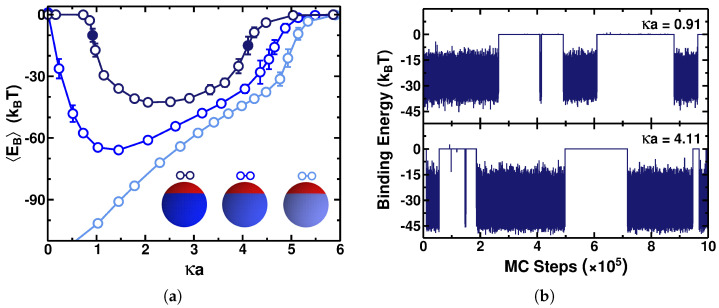
(**a**) Mean binding energy $\langle E_{B}\rangle$ as a function of κa for a PE chain containing N=50 monomers onto a sphere of radius a=70 Å and with the charge density given in Equation (2), with the patch sizes defined by the angles $\theta_{p}=\theta_{n}=120^{\circ }$. The parameters are $\sigma_{n}=0.03$ C/m^2^ and $\sigma_{p}=0.01$, 0.02, and 0.03 C/m^2^ for the light-blue, blue, and dark-blue curves, respectively. In the legend of panel (**a**) and hereafter, the intensity of the blue color for the spherical Janus-type particle is correlated with the magnitude of the respective surface charge density. (**b**) Trajectories of the variation in the binding energy $E_{B}\left(\mathrm{number}\;\mathrm{of}\;\mathrm{MC}\;\mathrm{steps}\right)$ with time recorded throughout the course of the simulations, plotted for the parameters corresponding to those of the filled blue symbols in panel (**a**).

Figure 2a presents $\langle E_{B}\rangle$ as a function of κa for three different charge densities and for a patch size defined by $\theta_{p}=\theta_{n}=120^{\circ }$. The light-blue curve in Figure 2a shows the results for the particle with a neutral net charge, while the blue and dark-blue ones refer to the particles with a positive net charge (the same charge as that of the PE chain). The light-blue curve exhibits a behavior analogous to that of the PE adsorption on a homogeneously oppositely charged surface [39] due to the attractive PE–particle interactions of a dipolar nature. At low salt concentrations, the ES attraction is strong enough to overcome the entropic loss due to the reduction of translational and conformational degrees of freedom of the PE chain caused by the adsorption process. As the salt concentration increases, the ES screening progressively weakens the attractive ES interactions that favors the emergence of configurations with the PE chain being desorbed, as already discussed in ref. [39]; see Figure 2a. At high enough ionic strengths, the mean binding energy nearly vanishes, $\langle E_{B}\rangle \approx 0$, and the adsorption is completely suppressed by strong screening.

In the situations when the charge density of the positive patch increases—see the blue and dark-blue curves in Figure 2a—the mean binding energy exhibits a nontrivial behavior. At low salt concentrations, $\langle E_{B}\rangle \approx 0$, characterizing the desorbed state of the PE, caused by the ES repulsion due to a nonzero net charge of the particle. In the regimes of higher salt concentrations, local attractive interactions become more and more important, leading to the PE–particle adsorption and to an increase in the magnitude of the mean binding energy. Consequently, after $\langle E_{B}\rangle$ reaches its minimal value (corresponding to the strongest PE–particle ES interactions because $E_{B}<0$), the behavior becomes analogous to that for PE adsorption onto a particle with an opposite net electric charge; see refs. [39,48].

The dark-blue curve in Figure 2a follows the free-PE-chain behavior until the data approach the boundary κa≈1, whereas the blue curve deviates at smaller κa values. This difference arises because the dark-blue curve in Figure 2a corresponds to a particle with a higher density of positive charges, requiring stronger ES screening in order to ensure the dominance of the local interactions between the positively charged PE and the negatively charged cap of the spherical particle.

Figure 2b illustrates the binding energy $E_{B}\left(\mathrm{number}\;\mathrm{of}\;\mathrm{MC}\;\mathrm{steps}\right)$ at each step of the Monte Carlo simulations at regimes of low and high ionic strength. These conditions are indicated by the filled points in the dark-blue curve in Figure 2a, at κa=0.91 and at κa=4.11, respectively. These points are situated within the range of κa where there is a coexistence of the adsorbed and desorbed states of the PE chain. The desorbed state is characterized by a binding energy nearly equal to zero, whereas in the adsorbed state, the binding energy fluctuates between ≈$-45\;k_{B}T$ and ≈$-10\;k_{B}T$. In the same way as that observed at high salt concentrations, we observe a desorption → adsorption first-order-like transition when the ES screening reaches the regime where the local attractive interactions become dominant over the repulsion stemming from the particle’s net charge. Figure 2b shows that the PE adsorption onto a more positively charged particle with $\sigma_{p}=0.03$ C/m^2^ (the dark-blue curve) is observed only when the value of κa reaches about unity, unlike for the particle that is less positively charged (see the blue curve in Figure 2a), in which a very small increase in the salt concentration from κ=0 leads to an increase in $|E_{B}|$ and, as a result, to a significant PE–particle adsorption.

### 3.2. Radius of Gyration of the Polymer

**Figure 3:**
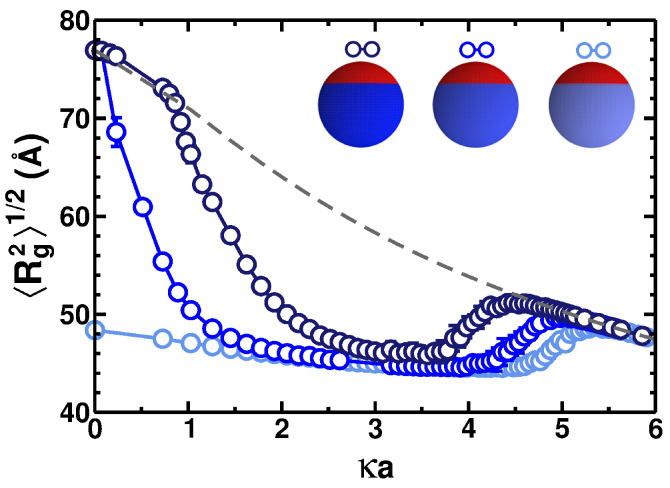
Radius of gyration $\sqrt{\langle R_{g}^{2}\rangle }$ as a function of κa for a PE chain of N=50 monomers. The color and symbol schemes are identical to those in Figure 2a. The gray dashed curve illustrates the results for a free PE chain in the absence of adsorbing spherical particle. As κ increases, the PE–PE ES repulsions (8) decrease, making the chain less extended, as shown. The particle has a patch size defined by the angles $\theta_{p}=\theta_{n}=120^{\circ }$ and the sphere radius is a=70 Å.

Figure 3 presents the root mean square radius of gyration of the PE chain $\sqrt{\langle R_{g}^{2}\rangle }$ as a function of κa for PE adsorption onto a particle with the same charge densities as in Figure 2a. We also use the same symbol and color schemes in these two figures for clarity of presentation. In the case of a neutral particle, at low salt concentrations, the PE chain has a radius of gyration much smaller than that expected for a free chain (see the gray dashed line in Figure 3), highlighting the dramatic reduction in the chain dimensions caused by adsorption onto the particle. With increasing salt concentration, the radius of gyration decreases initially because the screening of the monomer–monomer ES interactions makes fewer extended configurations of the PE being occupied in the space of configurations. This behavior is analogous to the PE adsorption onto the homogeneously charged spherical [39] and cylindrical [46] macroions as well as onto the Janus nanospheres [48].

The increase in the charge density of the positive patch—which leads to a more positive total charge of the particle—causes the positive PE chain to follow the free-chain behavior because the like-charge PE–particle ES repulsions prevent the PE–particle adsorption. This occurs at κa≈0 for the spherical particles with $\sigma_{p}=0.02$ C/m^2^ (the blue curve in Figure 3) or κa∼1 for the particle with $\sigma_{p}=0.01$ C/m^2^ (the dark-blue curve). This takes place, logically, in the same regimes of the ionic strengths in which the mean binding energy is nearly vanishing in Figure 2a. Increasing the salt concentration in Figure 3, the transition from the desorbed to the adsorbed states is accompanied by a decrease in the average radius of gyration due to a spatial confinement of the PE chain. Additionally, stronger screening of the ES interactions reduces the monomer–monomer repulsion along the PE, leading to less extended configurations of the PE chain.

The overall *nonmonotonic* dependence of $\sqrt{\langle R_{g}^{2}\left(n_{0}\right)\rangle }$ is a clear manifestation of a compactification transition at relatively low salinities and of a decompactification of the PE chain at considerably higher salt concentrations. The latter PE adsorption–desorption transition takes place in the range of κa≈4…5. Despite strong ES screening, which leads to overall more compact PE chain configurations, the increase in ${\langle R_{g}^{2}\rangle }^{1/2}$ reported at these κa values in Figure 3 is caused by the contribution of configurations in the desorbed state, having larger values of the radius of gyration.

### 3.3. Adsorption–Desorption Curve and Critical Charge Density

The discontinuous transitions shown in Figure 2b enable us to define a *critical* charge density of the negative patch, $\sigma_{n,c}$. The latter is defined when the fraction of the number of configurations in which the PE is in the adsorbed state is exactly 1/2. This criterion was also adopted in several of our previous studies, see, i.a., refs. [48,54,57] for the details on the methodology of determining the value of $\sigma_{c}$ based on the analysis of the simulated PE configurations.

**Figure 4:**
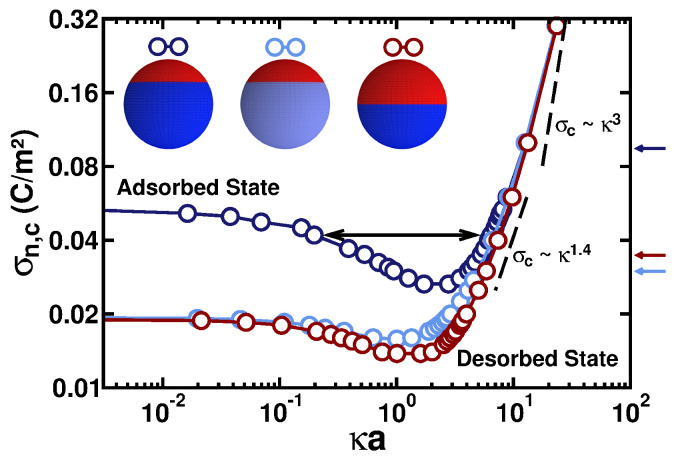
Magnitude of the critical surface charge density $\sigma_{n,c}$ as a function of κa for a PE chain with N=50 monomers. The particles represented by the dark-blue and light-blue curves have starting densities of $\sigma_{p}=0.03$ C/m^2^ and 0.01 C/m^2^, respectively, with $\theta_{p}=\theta_{n}=120^{\circ }$, whereas the particle represented by the dark-red curve has $\sigma_{p}=0.03$ C/m^2^ and $\theta_{p}=\theta_{n}=90^{\circ }$ (see the legend in the plot). The arrows of the respective colors on the right axis indicate (for the $\sigma_{c}\left(\kappa a\right)$-data presented) the densities where the net charge of the respective particles (without the adsorbed PE chain) is equal to zero; that is, net-neutral Janus-type particles. The scaling relations of the type (1) at intermediate and large κa values are indicated as the dashed lines. For the black-circle data, the region of κa indicated by the double arrow shows the critical charge densities at which—upon increasing the value of κa — the desorbed-to-adsorbed and the adsorbed-to-desorbed transitions take place (see also the description in the text).

Figure 4 shows $\sigma_{n,c}$ as a function of κa for two values of the positive charge densities, namely $\sigma_{p}=0.03$ C/m^2^ (the dark-blue curve) and $\sigma_{p}=0.01$ C/m^2^ (the light-blue curve), computed with the same patch size as in Figure 2. As indicated in Figure 4, the region above the curves comprises the values of $\sigma_{n}$ and κa at which the PE chain is adsorbed to the patchy particle, while in the regions below the respective critical curves, stable adsorption is not observed. As seen in Figure 4, for the region of small solution salinities of κa≲1…3, when the PE chain mostly experiences the charge of the entire particle, its net charge is particularly important as a limiting factor of adsorption; in contrast, for κa≫1, the PE interacts mainly *locally* and the long-range ES interactions are strongly screened. Thus, the charge density of the negative patch needed to have adsorption is large, in order to compensate for the repulsions stemming from the positive portion of the spherical particle.

At low ionic strengths, the critical surface charge density $\sigma_{n,c}$ of the ES–attractive negative cap reaches a value of $\sigma_{n,c}\approx 0.05$ C/m^2^ for $\sigma_{p}=0.03$ C/m^2^ and of $\sigma_{n,c}\approx 0.02$ C/m^2^ for the situation $\sigma_{p}=0.01$ C/m^2^, respectively. These situations for the positive cap charge densities are considered in Figure 4. As expected, smaller ES–attractive negative spherical caps should have a larger magnitude of the surface charge density in order to adsorb the positively charged PE chain, which is being ES repelled from the larger positive cap on the opposite side of the particle. For both values of $\sigma_{p}$ considered, the computed critical values of $\sigma_{n,c}$ are *smaller* than the negative surface charge densities needed to fully neutralize the particle (see the respective arrows for the densities that would produce net-neutral particles on the right side of Figure 4). We thus observe the complexation of the PE onto a *likely charged* particle. A similar physical phenomenon is observed for the PE–protein complexation on the “wrong side” of the isoelectric point [82,87].

The effect of a variable patch size is now examined; see the dark-red curve in Figure 4 plotted for the parameters $\theta_{n}=90^{\circ }$ and $\sigma_{p}=0.03$ C/m^2^. The critical charge density $\sigma_{n,c}$ needed to trigger the PE adsorption onto a particle with these properties is similar to what we have obtained for the situation $\sigma_{p}=0.01$ C/m^2^ and $\theta_{n}=120^{\circ }$. The decrease in the positively charged area of the particle is thus compensated by the increasing of the surface charge density $\sigma_{p}$, as intuitively expected. In particular, for small κa values, the particles with a *large positively charged cap*—which is ES repelling the positively charged PE chain—require much *larger* values of surface charge density on the *smaller negatively charged* cap. The latter is the one eventually attracting the PE and triggering the ES-driven adsorption.

As a support of this statement, the reader should compare the low-salt behavior of the dark-blue and dark-red curves in Figure 4. In these cases, the value of $\sigma_{n,c}$ decreases with increasing κa, and $\sigma_{n,c}$ reaches a *minimum* at κa≈1.5; see Figure 4. Under these conditions, the Debye screening length becomes comparable to the size of the negative patch of the particle, i.e., $\kappa^{-1}∼a$, thus reducing the influence of particle’s net charge.

All of the curves in Figure 4 converge (at a fixed *a* value) for κa≫1 to the state dominated by the *local* ES interactions; this is the regime of strongly screened ES. In this regime, we recover the behavior observed for the positively charged PE adsorbed onto a homogeneously negatively charged planar surface, with the known scaling relation $\sigma_{n,c}\left(\kappa \right)∼\kappa^{3}$ [5,41]. For an experimental range of salinities and particle dimensions, with a typical range of 5≲κa≲15 [33,74,75], the scaling relations predicted from our simulations are in agreement with the ones observed for the PE complexation with several types of spherical colloids, namely $\sigma_{c}\left(\kappa \right)∼\kappa^{1.4}$, as indicated in Figure 4 by the dashed line.

We stress here that the nonmonotonic behavior of $\sigma_{n,c}$ versus κa presented in Figure 4 gives rise to the existence of *two phase transitions* in our computer simulations. Namely, with an increasing κa value, the PE goes from the desorbed to the adsorbed state at some critical surface charge density $\sigma_{n,c}$, then at a larger κa value and at the same $\sigma_{n,c}$ value, a transition from the adsorbed to the desorbed state takes place. Note that this nonmonotonic $\sigma_{n,c}\left(\kappa a\right)$–behavior emerges for the adsorption of only *similarly charged* PE onto a spherical particle (for the *oppositely charged* PEs and surfaces of any geometries, the critical surface charge density always *increases* with κa [41]). For the PE adsorption onto a net-neutral Janus particle treated in ref. [48], a monotonic behavior of $\sigma_{c}$ versus κa was also observed.

Also note here that the so-called resolubilization or decondensation at comparatively high concentrations of the added salt is a well-studied phenomenon for the complex formation of DNA molecules with a number of DNA-condensing cations and (at least locally) positively charged proteins [25,111,112,113].

### 3.4. Implications of Low-Dielectric Interfaces

Now, we discuss the main new aspect of the current study, namely the effects of a low-dielectric interior of patchy heterogeneously charged particles on the characteristics of critical PE–particle adsorption.

The low-$\epsilon_{\mathrm{part}}$ interior of homogeneously charged surfaces causes a *weaker* PE adsorption due to the ES-induced repulsion of the PE monomers from their image charges [114] being formed underneath the adsorbing interface; see refs. [43,61] for the investigation of these effects on the PE adsorption. For the PE adsorption onto a patchy particle, in addition to the ES potential of the polarization charges given in Equation (10), the potential due to the particle-charge distribution described in Equation (4) is also affected by a different/low value of the dielectric constant under the adsorbing boundary; see Equation (4). We incorporated these effects of a low-dielectric interior in the model and performed the respective computer simulations of PE adsorption onto such spherical low-dielectric patchy particles.

**Figure 5:**
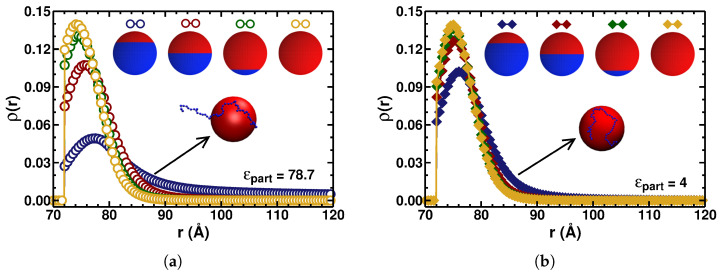
Density distribution of the PE monomers under the conditions *above* the adsorption–desorption transition as a function of the distance from the center of a particle with a dielectric constant of (**a**) $\epsilon_{\mathrm{part}}=78.7$ (empty circles) and (**b**) $\epsilon_{\mathrm{part}}=4$ (filled diamonds). The particle radius is a=70 Å, the surface charge densities are $\sigma_{n}=\sigma_{p}=0.03$ C/m^2^. For the dark-blue, dark-red, dark-green, and dark-yellow curves, the patch sizes are defined by $\theta_{p}=\theta_{n}=\{120^{\circ }$, $90^{\circ }$, $45^{\circ }$, $0^{\circ }\}$, respectively, as indicated in the legends. The salt concentration in the solution is fixed at 5 mM (corresponding to κa=1.625, valid for the curves of all colors presented in these plots).

Figure 5 presents the monomer-density distribution as a function of the distance from the particle center for four different patch sizes with $\theta_{p}=\theta_{n}$ (i.e., no neutral region on the spherical particle, see Figure 1a). In Figure 5a, we show the case without a dielectric interface; that is, $\epsilon_{\mathrm{part}}=\epsilon_{\mathrm{water}}=78.7$, while in Figure 5b, the patchy spherical particle has a low internal dielectric constant of $\epsilon_{\mathrm{part}}=4$. Thus, it is possible to separately analyze the influence of the charge-patch size and of the internal dielectric constant on the PE–particle adsorption.

Starting with the case of the homogeneously charged sphere—i.e., $\theta_{p}=\theta_{n}=0$, see the dark-yellow curves in Figure 5a,b—the distribution of the PE monomers in the presence of a dielectric interface/boundary is slightly less confined compared to the case with a uniform-dielectric medium (compare the dark-yellow curves in panels (a) and (b) of Figure 5). As mentioned above, this is caused by the repulsive potentials of the polarization charges, similar to the trends predicted in ref. [43] based on the analytical WKB-type computations of the adsorption onto low-dielectric interfaces. The leading-order term of the ES potential of the particle (see the l=0 term in Equation (4)) does not change with variations in the internal dielectric constant. The potential due to the polarization charges (given in Equation (10)) is responsible for a *decrease* in the binding affinity, causing the PE configurations be *less confined*, as compared to those for the PE–particle interactions for the situation without a dielectric interface.

As a positively charged region on the surface of the patchy spherical particle *increases* in size (i.e., when the values of $\theta_{p}=\theta_{n}$ increase; see the caption of Figure 5), the PE-monomer distribution becomes *less* confined close to the surface due to the ES-driven repulsion from a positively charged surface patch. When the dielectric constant of the particle is much smaller than that of the solvent, $\epsilon_{\mathrm{part}}\ll \epsilon_{\mathrm{water}}$, a more attractive ES potential generated by the particle overcomes the repulsion of the PE monomers from their image charges. This results in a more confined adsorbed PE chain than in the scenario with no dielectric interfaces.

**Figure 6:**
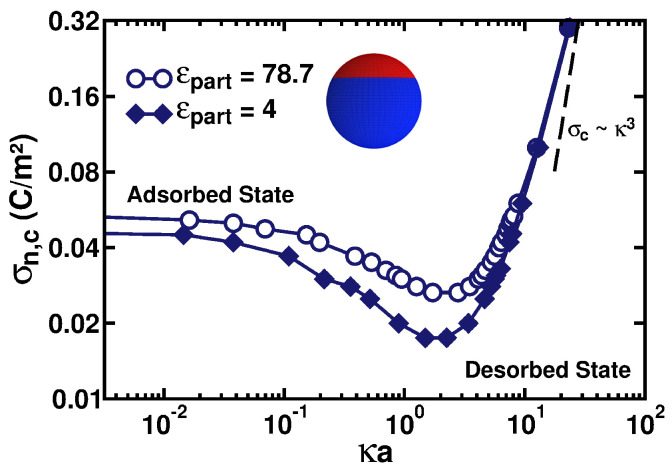
Critical surface charge density of the negative patch $\sigma_{n,c}$ plotted as a function of κa for a PE chain with N=50 monomers. The values of the parameters are as follows: the positive charge density is $\sigma_{p}=0.03$ C/m^2^, the patch sizes are defined by $\theta_{p}=\theta_{n}=120^{\circ }$, and the sphere radius is a=70 Å. The empty dark-blue circles represent a particle with a dielectric constant equal to that of the medium, while the filled dark-blue diamonds are for the situation $\epsilon_{\mathrm{part}}\ll \epsilon_{\mathrm{water}}$; see the legend.

Figure 6 presents the critical negative charge density $\sigma_{n,c}$ as a function of κa for a particle with the patch sizes defined by $\theta_{p}=\theta_{n}=120^{\circ }$. The computed adsorption–desorption demarcation boundaries demonstrate that it is possible to also see a significant attraction of the PE chain to the particle with a low-dielectric core; compare the empty circles and filled diamonds in Figure 6. Despite a repulsive ES contribution of the induced polarization charges in the scenario $\epsilon_{\mathrm{part}}\ll \epsilon_{\mathrm{water}}$, a stronger attractive contribution emerges due to the existence of a region of the particle with a *similar* charge to that of the PE chain. Therefore, we do find from the simulations that somewhat *smaller* negative surface charge densities are needed to promote the ES-driven adsorption of the positively charged PE chain in this case. The effect is not dramatic, but the differences we detect in the respective $\sigma_{n,c}$ values are clear and systematic; these differences are in the same direction in the entire range of the salt concentrations considered in our simulations (the empty circles are above the filled diamonds in Figure 6).

**Figure 7:**
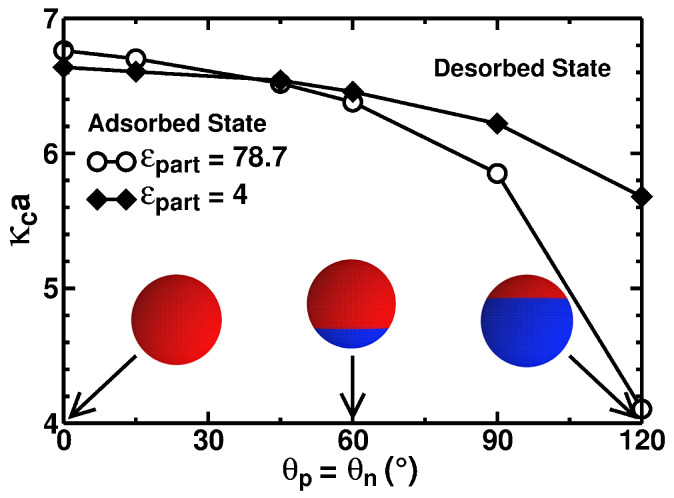
Critical dimensionless solution-salinity parameter $\kappa_{c}a$ plotted as a function of the patch dimensions on the spherical particle. The charge densities of the spherical caps are *fixed* at $\sigma_{p}=\sigma_{n}=0.03$ C/m^2^. The empty black circles represent a particle with a dielectric constant equal to that of the medium, while the filled black diamonds are for the situation $\epsilon_{\mathrm{part}}\ll \epsilon_{\mathrm{water}}$.

Figure 7 presents the critical values of $\kappa_{c}a$ as a function of the patch-controlling angles $\theta_{p}=\theta_{n}$ for the cases with and without the dielectric interface. This time, for the *fixed* $\sigma_{n,p}$ values of the charge densities of the spherical caps and for *varying* patch angles, we conducted systematic computer simulations to rationalize the critical-adsorption conditions. From the simulation data, however, the values of critical $\kappa_{c}a$ are now being extracted (for the situation of fixed surface charge densities of the caps). The criterion to calculate the critical $\kappa_{c}$ values here is identical to that used to enumerate the $\sigma_{n,c}$ values in Figure 4. Namely, it is a 50%–50% distribution between the adsorbed and desorbed conformations of the polymer, as recorded during the simulation time. These cases are shown by the filled diamonds and empty circles in Figure 7, respectively. We remind the reader here that the adsorbed state exists for the conditions below the shown adsorption–desorption boundary curves, while the desorbed state is above them.

We observe that as $\theta_{p}=\theta_{n}$ decreases and the Janus-type patchy particle effectively approaches a homogeneously charged sphere, the PE chain experiences a weaker attraction to the particles with a *low* dielectric constant inside them due to the repulsive contribution of the polarization charges, as given in Equation (10). This is evidenced by a somewhat lower ionic concentration needed to promote the PE desorption for the case with $\epsilon_{\mathrm{part}}=4$ (compare the values of $\kappa_{c}a$ on the left side of Figure 7). As the size of the positive patch increases, this behavior gets reverted at $\theta_{p}=\theta_{n}∼45^{\circ }$, and the critical $\kappa_{c}a$ value becomes substantially larger for the case of the particle with the low-dielectric interior (compare for this the values of $\kappa_{c}a$ on the right side of Figure 7).

This can be understood as follows: the ES potential of the polarization charges $\Psi_{\mathrm{polar}}$ given by Expression (10) generated by the presence of a monomer close to the particle with a low dielectric constant is always repulsive and is independent of the particle charge distribution. When the particle is homogeneously charged, carrying a charge opposite to that of the adsorbing PE chain, the ES potential $\Psi_{\sigma }$ is attractive (largely independent of the internal dielectric constant of the particle). The sum of these two contributions for the case when the particle has a low dielectric constant leads to *weaker* adsorption compared to the case when the dielectric interface is absent, i.e., when $\Psi_{\mathrm{polar}}=0$, as can be seen in Figure 7 for $\theta_{p}=\theta_{n}=0$. With an increase of $\theta_{p}=\theta_{n}$, the emergence of a patch similarly charged to the PE on the particle surface causes $\Psi_{\sigma }$ to increase (i.e., for the convention of the signs chosen, the potential $\Psi_{\sigma }$ becomes less negative). Importantly, this variation in the potential is more intense for the case when the particle has a high dielectric constant. This variation for a high-dielectric case *compensates* for the summation of the repulsive contribution of the polarization charges in the low-dielectric case, and for $\theta_{p}=\theta_{n}>45^{o}$ (see Figure 7), the total potential in the low-dielectric case becomes *more attractive* to the PE chain.

## 4. Discussion, Conclusions, and Perspectives

In this work, we investigated the characteristics of the adsorption of a positively charged PE chain onto spherical Janus-type particles featuring patches of both negative and positive charges on their surface. The particular focus was on the effect of a dielectric interface between such a patchy particle and the surrounding solvent. A key finding was the observation of two distinct adsorption–desorption transitions for PE interacting with particles bearing a net charge of the same sign. This phenomenon can be discussed in terms of either an energetic or a conformational transition. These transitions were found to be characterized by abrupt changes in the mean binding energy, reflecting the dynamical transitions between the adsorbed (more compact) and desorbed (more extended) states of the PE. The maximum of the PE–particle binding affinity was found to occur at κa≈1.5, i.e., when the Debye length is comparable to the size of the negative patch, $\lambda_{D}=1/\kappa \approx 46.7$ Å for the particle radius a=70 Å used in the simulations.

The presence of a dielectric interface leads to *enhanced* PE adsorption onto patchy particles. Note that earlier theoretical results for the PE adsorption onto *homogeneously* charged particles with a low-dielectric interior have predicted a *reduction* in PE adsorption [43]. However, as the particle approaches a uniformly charged sphere, this trend of the enhanced adsorption reverses, in agreement with the previous findings in the literature [28,61]. The enhanced adsorption observed here in the situation of heterogeneously charged Janus-type spherical particles can be attributed to a competition between the more attractive effects generated by a patchy particle with a low internal dielectric constant and the repulsion of the PE monomers from their image charges. Regarding the effects of the dielectric-constant mismatch, a recent study [60] has also demonstrated an increased binding affinity of the PEs to oppositely charged surfaces bearing discrete charge patterns. In the study [60], the localized charged sites acted as “bridges” between the monomers and their image charges; an effect further amplified by a dielectric mismatch.

In general, any distribution of patches of electric charges in terms of their size and geometric arrangement is possible on the spherical surface within the current model. This variability from the ES viewpoint, plus the low-dielectric particle interior—one of the main novelty of this study—is ultimately needed to apply such a model *quantitatively* to the real-world scenarios of PE–protein ES-driven complexation and solubilization (as in the works of Dubin mentioned in the introduction). As any charge distribution can be treated in terms of the resulting ES potential, the PDB data for a given globular protein can be directly imported into the ES model of this type. This is, evidently, our next step in this direction. Further steps into a “terra incognita” include the issues of accommodating more generalized/sophisticated conditions, such as some particular multivalent ions, the impact of a pH-regulated charged groups, implications of the charge regulation, and more realistic (non-ideally spherical) protein geometries.

**Figure 8:**
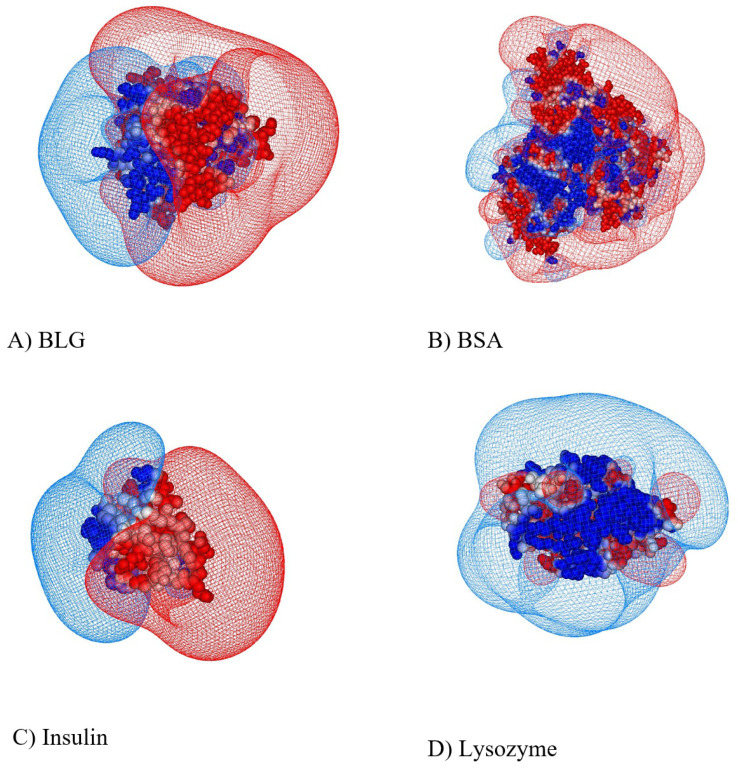
Distribution of the ES potential on the surface of four abundant dipolar-type protein molecules at pH 7 and ionic strength of 0.15 M for bovine serum albumin (BSA), lysozyme, insulin, and β-lactoglobulin (BLG). Reprinted with permission from ref. [76]. Copyright 2003 American Chemical Society.

From the perspective of applications, one can highlight the agreement of our observed trends with the experimental results of the *nonmonotonic* behavior for the binding affinity as a function of the salt concentration observed for heparin–antithrombin [81] and heparin–insulin [76] complexes. As mentioned in Section 1.2, the latter study examined the properties of the interactions between the polyanion heparin and insulin protein. Insulin is a type of globular protein and contains clearly defined positively and negatively charged caps on its surface; see Figure 8. The authors of ref. [76] have demonstrated the existence of transitions between the complexed/compact and noncomplexed/decondensed states at a given critical value of the pH, namely at pH_c_ [76].

The variation in the ionic strength was shown to exhibit *nonmonotonic* behavior of the pH_c_ as a function of κ; see Figure 2 in ref. [76]. The maximal pH value was shown to be attained when the Debye length 1/κ became of the order of the protein radius (κa≈1). We observed a very similar trend for the critical-adsorption condition of $\sigma_{n,c}$, as reported in Figure 4 and Figure 6. The current study thus enables one to extend this analytical and simulational approach to more realistic systems of, e.g., numerous important PE–protein complexes, as mentioned in Section 1.1.

One more example of the role of the ES interactions in certain biological functions of some nucleic acid-binding proteins is the M2-1 protein of the human Respiratory Syncytial Virus (hRSV) [115]. M2-1 is a transcriptional anti-termination factor of the viral polymerase and its globular domain displays a heterogeneous ES-potential surface, including a positively charged patch, playing a key role in the interactions that M2-1 establishes with RNA [115].

Another example involves the so-called cold-shock proteins (Csp), acting to preserve cell viability at low temperatures by binding to nucleic acids and thus modulating the gene expression. Caruso et al. [116] recently demonstrated that the CspA of *Corynebacterium pseudotuberculosis* also exhibits an asymmetric surface of the ES potential, containing a positively charged patch identified as the primary site of interactions with the single-stranded DNA (Y-box sequence).

The role of image charges quantified here can also enable a more detailed analysis and, hopefully, serve for a deeper interpretation of the data on PE–protein complexation, i.a., at varying solution salinities. The effects of image charges are of importance both for wrapping and complexation of very flexible PEs such as single-stranded DNA/RNA (i.a., upon their wrapping [23,117] around single-wall carbon nanotubes [118,119]) and PAA [28] molecules, for intermediately flexible PEs such as Na-PSS, PDADMAC, and hyaluronic acid [28,74,77], as well as for semiflexible or rigid (depending of the external salinity) PEs such as the double-stranded DNA in its canonical high-humidity B-form.

Finally, the current model can include two types of specificity to study specific systems. The first one is regarding the charge-regulation effects, recognized to be important for PE–protein interactions [26,38]. These effects can result in an induced net charge or in a modification of the charge distribution of a protein due to the presence of the vicinal PE chain. To achieve this, a specific spatial distribution of the protein residues and of their respective pK_*a*_ values must be considered. Another specificity is the “structural” persistence length of the PE, in addition to the ES PE persistence already considered in the present simulations. This can be achieved by including a force field with the parameters dependent on a specific PE molecular structure.

## Data Availability

All data used in this study appear in the submitted article.

## Abbreviations

The following abbreviations are used in this manuscript:

PE	polyelectrolyte
ES	electrostatic/electrostatics
WKB	Wentzel–Kramers–Brillouin

## Appendix A. ES Potential of a Patchy Particle

The dimensionless ES potential Ψ obeys the Laplace equation inside the particle ($\Psi =\Psi_{\mathrm{part}}$ for r≤a) and the linearized Poisson–Boltzmann equation in the external electrolyte solution ($\Psi =\Psi_{\mathrm{water}}$ for r>a), i.e.,

(A1) $$\nabla^{2}\Psi_{\mathrm{part}}\left(r,\theta \right)=\;0\;and\;\nabla^{2}\Psi_{\mathrm{water}}\left(r,\theta \right)\;=\;\kappa^{2}\Psi_{\mathrm{water}}.$$

The Equation (A1) consist of a set of two second-order differential equations to be solved, subject to an appropriate set of four boundary conditions. Two of these conditions demand a finite potential $\Psi_{\mathrm{part}}$ at r=0 and a vanishing $\Psi_{\mathrm{water}}$ at infinity. Then, we can write the following expansions [114]:

(A2) $$\Psi_{\mathrm{part}}\left(r,\theta \right)\;=\;\sum_{l=0}^{\infty }A_{l}r^{l}P_{l}\left(\cos\theta \right)\;\mathrm{and}\;\Psi_{\mathrm{water}}\left(r,\theta \right)=\sum_{l=0}^{\infty }B_{l}k_{l}\left(\kappa r\right)P_{l}\left(\cos\theta \right).$$

The remaining two conditions are related to the continuity of both the potentials and the electric displacements at the interface between the particle interior and the electrolyte,

(A3) $$\Psi_{\mathrm{water}}\left(r=a,\theta \right)=\Psi_{\mathrm{part}}\left(r=a,\theta \right)$$

and

(A4) $$\epsilon_{\mathrm{water}}\frac{\partial \Psi_{\mathrm{water}}}{\partial r}{|}_{r=a}-\epsilon_{\mathrm{part}}\frac{\partial \Psi_{\mathrm{part}}}{\partial r}{|}_{r=a}=-\frac{e_{0}\sigma \left(\theta \right)}{\epsilon_{0}k_{B}T}.$$

Upon applying (A3) and (A4), we obtain $A_{l}=B_{l}k_{l}\left(\kappa a\right)/a^{l}$ and

(A5) $$\sum_{l=0}^{\infty }B_{l}P_{l}\left(\cos\theta \right)\left[\epsilon_{\mathrm{water}}\kappa k_{l}^{\prime }\left(\kappa a\right)-\epsilon_{\mathrm{part}}\frac{lk_{l}\left(\kappa a\right)}{a}\right]=-\frac{e_{0}\sigma \left(\theta \right)}{\epsilon_{0}k_{B}T}.$$

In Equation (A1), we neglect the effects of high salt concentrations onto the form of the right-hand side of the Poisson–Boltzmann equation; see refs. [120,121]. We can find the solution to Equation (A5) by using the orthogonality conditions of the Legendre polynomials [98,114], i.e., $\int_{-1}^{1}P_{m}\left(x\right)P_{n}\left(x\right)dx=2\delta_{mn}/\left(2m+1\right)$, where $\delta_{mn}$ denotes the Kronecker δ-symbol. Then, for an arbitrary σ(θ) distribution (2), the coefficients are as follows:

(A6) $$B_{l}=\frac{e_{0}}{2\epsilon_{0}k_{B}T}\frac{(2l+1)a}{\epsilon_{\mathrm{part}}lk_{l}\left(\kappa a\right)-\epsilon_{\mathrm{water}}\kappa ak_{l}^{\prime }\left(\kappa a\right)}\int_{0}^{\pi }\sigma \left(\theta \right)P_{l}\left(\cos\theta \right)\sin\theta d\theta .$$

The ES potential $\Psi_{\mathrm{water}}\left(r,\theta \right)$ in Equation (A2) with the expansion coefficients (A6) is exactly the surface-charge-induced ES potential $\Psi_{\sigma }\left(r,\theta \right)$ in Equation (4) of the main text.

## Appendix B. ES Potential of the Polarization Charges at the Particle Surface

The ES potential of the polarization charges—emerging due to the presence of the *j*th-monomer of the PE close to the particle—is obtained from the ES potential of a charge $q_{j}$ in an electrolyte solution in the presence of a dielectric sphere. In this case, the ES potentials inside and outside of the particle are given by (A1), with the addition of the ES potential from the monomer itself at the external region. As the potential should be finite at r=0, and to vanish at infinity, we can write the expansions

(A7) $$\begin{array}{cc} \Psi_{\mathrm{part}}\left(r,\theta ,\phi \right) & =\sum_{l=0}^{\infty }\sum_{m=-l}^{+l}A_{lm}r^{l}Y_{lm}\left(\theta ,\phi \right) \end{array}$$

and

(A8) $$\begin{array}{cc} \Psi_{\mathrm{water}}\left(r,\theta ,\phi \right) & =\sum_{l=0}^{\infty }\sum_{m=-l}^{+l}B_{lm}k_{l}\left(\kappa r\right)Y_{lm}\left(\theta ,\phi \right)+\frac{l_{B}z_{j}}{e_{0}}\frac{e^{-\kappa |\vec{r}-\vec{r_{j}}|}}{|\vec{r}-\vec{r_{j}}|}, \end{array}$$

where $Y_{lm}\left(\theta ,\phi \right)$ are the standard spherical harmonics [114]. The screened Coulomb potential can be written as the following expansion [122]

(A9) $$\frac{e^{-\kappa |\vec{r}-\vec{r_{j}}|}}{|\vec{r}-\vec{r_{j}}|}=\sum_{l=0}^{\infty }\sum_{m=-l}^{+l}4\pi \kappa k_{l}\left(\kappa r_{j}\right)i_{l}\left(\kappa r\right)Y_{lm}\left(\theta ,\phi \right)Y_{lm}^{*}\left(\theta_{j},\phi_{j}\right),$$

where $i_{l}\left(x\right)$ is the modified spherical Bessel functions of the first kind.

The potentials (A7) and (A8) are subject to the continuity of both the potentials themselves and of the electric displacements at the interface between the particle interior and the electrolyte solution. Applying these boundary conditions,

(A10) $$\begin{array}{cc} \Psi_{\mathrm{water}}\left(r=a,\theta \right)=\Psi_{\mathrm{part}}\left(r=a,\theta \right)\;\mathrm{and}\;\epsilon_{\mathrm{water}}\frac{\partial \Psi_{\mathrm{water}}}{\partial r}{|}_{r=a} & =\epsilon_{\mathrm{part}}\frac{\partial \Psi_{\mathrm{part}}}{\partial r}{|}_{r=a}, \end{array}$$

yields for the coefficients $B_{lm}$ the result

(A11) $$B_{lm}=\frac{4\pi l_{B}z_{j}\kappa k_{l}\left(\kappa r_{j}\right)}{e_{0}}\left[\frac{\epsilon_{\mathrm{water}}\kappa ai_{l}^{\prime }\left(\kappa a\right)-\epsilon_{\mathrm{part}}li_{l}\left(\kappa a\right)}{\epsilon_{\mathrm{part}}lk_{l}\left(\kappa a\right)-\epsilon_{\mathrm{water}}\kappa ak_{l}^{\prime }\left(\kappa a\right)}\right]Y_{lm}^{*}\left(\theta_{j},\phi_{j}\right).$$

The ES potential of the polarization charges is given by the first term in Equation (A8). Their final form is obtained by substituting (A11) into expression (A8) and by employing the so-called “addition theorem” for the spherical harmonics [98,114]

(A12) $$P_{l}\left(\cos\theta_{rj}\right)=\frac{4\pi }{2l+1}\sum_{m=-l}^{+l}Y_{lm}\left(\theta ,\phi \right)Y_{lm}^{*}\left(\theta_{j},\phi_{j}\right),$$

which results in Equation (10) of the main text.
